# Impacts of Skin Eccrine Glands on the Measured Values of Transepidermal Water Loss

**DOI:** 10.7759/cureus.32266

**Published:** 2022-12-06

**Authors:** Hailey Schwab, Jamie Flora, Harvey N Mayrovitz

**Affiliations:** 1 Osteopathic Medicine, Nova Southeastern University Dr. Kiran C. Patel College of Osteopathic Medicine, Fort Lauderdale, USA; 2 Medical Education, Nova Southeastern University Dr. Kiran C. Patel College of Allopathic Medicine, Fort Lauderdale, USA

**Keywords:** hyperhidrosis, scopolamine, skin barrier, sweat gland, eccrine gland, sweat water loss, tewl, transepidermal water loss

## Abstract

Transepidermal water loss (TEWL) is widely used to assess and quantify skin insensible water loss to assess skin’s barrier function integrity. Low TEWL values are normally indicative of intact skin and a healthy functional barrier, whereas an increased TEWL reveals a disturbed or disrupted skin barrier. Because most skin sites at which these measurements are made have eccrine glands present, the contribution of the sweat gland activity to these measurements is variable and, in most cases, unknown. The separation between the contribution of water loss that is reflective of the skin barrier integrity versus that contributed via eccrine activation is not easy and is made more difficult since both components increase with increasing skin and environmental temperature. Endogenous factors that impact eccrine sweat gland activity include sympathetic nervous system activity, emotional stress, physical activity, eccrine gland density, and age. Exogenous factors that impact eccrine gland activity include ambient temperature and humidity and the climate where one resides. The aforementioned variables impact eccrine gland activity positively or negatively and therefore alter TEWL values accordingly. Although it may be theoretically possible to control all these factors, the difficulty in doing so results in only a few being controlled during most TEWL measurements. Such confounding processes may have impacted historical TEWL reference ranges and values previously reported. Thus, the impact of eccrine activation on standardly measured TEWL values is at this juncture unclear and may be a component contributing to some reported variability in TEWL values. To help clarify the issues, a literature review was conducted to investigate and summarize relevant prior research efforts and outcomes with respect to ways to consider eccrine activity in TEWL measurements and estimate the contribution of eccrine gland activity to TEWL values. Online databases such as Excerpta Medica Database (EMBASE), Public/Publisher Medline (PubMed), Elton B. Stephans Company (EBSCO), Google Scholar, and Wiley Online Library were searched with “transepidermal water loss” or “TEWL” in the title combined with “eccrine glands” or “sweat” anywhere in the text. The present findings indicate a multiplicity of biological and environmental variables impacting eccrine gland activity and thereby potentially affecting measured TEWL values. Even if laboratory conditions adhere to various guidelines and recommendations, it is not yet possible to separate the eccrine activation component from the parameter of true interest in the assessment of the skin’s physiological barrier function except for full gland deactivation. The amount that such eccrine gland activation impacts the measured value of TEWL is generally not determined using currently available methods and the only sure way to eliminate a confounding effect is to inactivate the glands during such TEWL measurements. Because such eccrine gland deactivating approach is not usually desirable or even possible, other approaches would be recommended. One would be the development of a measuring device that could distinguish between the component of TEWL that is associated with the skin barrier function and the other that is attributable to sweat gland activation. Further research and development along these lines appear warranted.

## Introduction and background

Transepidermal water loss (TEWL) is widely used to assess and quantify skin insensible water loss often to assess skin’s barrier function integrity [1-5]. Most measurement methods use devices that are placed in contact with skin and collect water vapor flux into a chamber for either a fixed amount of time or until a certain volume has been accumulated [1,6]. Devices are broadly characterized as having open or closed chambers [4,6,7]. The resulting measurement yields localized skin-related total water loss (TWL) that is composed of two components [1,2]. One component is attributable to water diffusion through the intact epidermis that depends on the epidermal-to-air gradient in water vapor pressure [1,2]. It is this epidermal water loss (EWL) component that is most directly related to the skin barrier function [1,2].Low TEWL values are normally indicative of intact skin and a healthy functional barrier whereas an increased TEWL reveals a disturbed or disrupted skin barrier [8]. However, because most skin sites at which these measurements are made have eccrine glands present, the contribution of the sweat gland activity to these measurements is variable and, in most cases, unknown. This sweat gland-related component is herein defined as sweat water loss (SWL). Because SWL is not intrinsically related to skin barrier function, its presence contributes to uncertainty in the assessment of the measurement of the integrity of the skin’s barrier function [9-11]. The separation between contributions of the EWL component that is reflective of the skin barrier integrity vs. that contributed via eccrine activation (SWL) is not easy. It is made more difficult since both components increase with increasing skin and environmental temperature [10,12-14]. EWL is diffusion driven and dependent on the gradient in water vapor pressure which itself increases with skin temperature [1,10,11,15]. Eccrine gland activation increases with increasing skin temperature with an indeterminate skin threshold temperature between 30°C and 36°C, thereby affecting the SWL component [1,10]. In addition, beyond local skin temperature triggering sweat gland activation, there are also a variety of systemic activators [16-20]. These include cholinergic agonists, sympathetic nervous system activation, emotional stress, and various medical conditions such as hyperhidrosis and cystic fibrosis [10,21-24]. These conditions and processes affect eccrine gland activation and thus cause variable effects on TEWL measurements. Such confounding processes may have impacted historical TEWL reference ranges and values previously reported [17,18] and other TEWL-related findings including the suggestion of the absence of a correlation between TEWL and skin’s barrier function [9,10,18]. Thus, the impact of eccrine activation on standardly measured TEWL values is at this juncture unclear and may be a component contributing to some reported variability in TEWL values [1]. Obtaining an accurate TEWL reading, without the contribution from sweat glands, has been attempted using several approaches [2,25]. These include avoiding sites where there is increased eccrine gland activity in the lower limbs and volar forearm, using in vitro methods instead of in vivo measurements, and attempting to inactivate eccrine glands [2,25]. These methods and related issues regarding eliminating the sweating component of TEWL are further explored in the present review with the aim of summarizing known impacts [1].

## Review

The purposes of this review were to (1) investigate and summarize relevant prior research efforts and outcomes with respect to ways to consider eccrine activity in TEWL measurements and (2) estimate the contribution of eccrine gland activity to TEWL values.

Eccrine sweat gland physiology and processes

**Figure 1:**
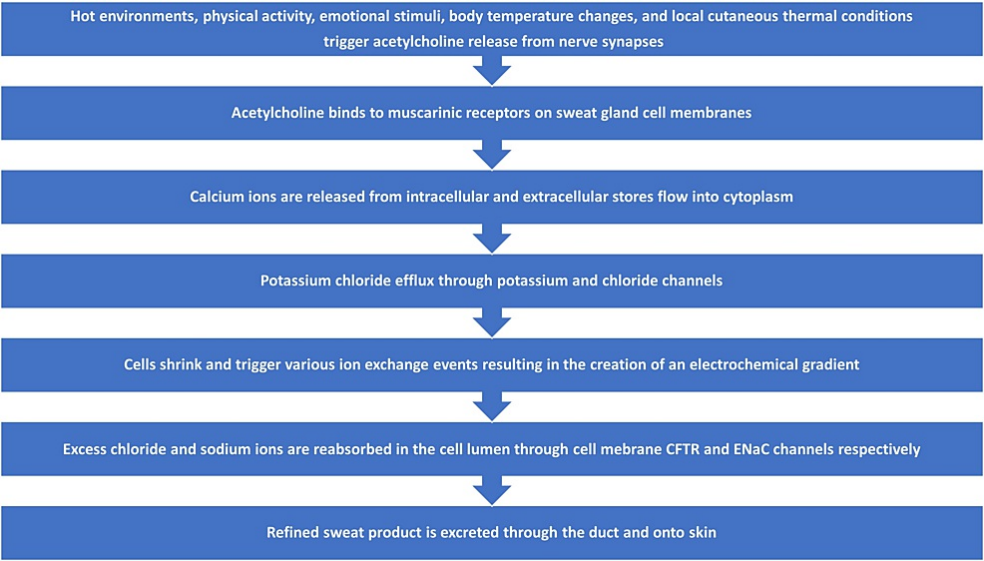
Overview of sweat gland activation, secretion, and excretion process The diagram illustrates the main aspects of the eccrine sweat gland activation, secretion, and excretion process. CFTR = cystic fibrosis transmembrane conductance regulator, ENaC = epidermal sodium channels Reference: [9] Author's own creation.

The sequence of events involved in the process of sweat gland activation, secretion, and excretion is summarized and depicted in Figure 1. The eccrine gland is the most prevalent sweat gland, and it is present in the dermis layer of the skin in various densities at different anatomical locations [26-29]. The two main parts of an eccrine gland are the secretory coil and the duct [26]. The secretory coil contains three cell types: clear cells that primarily secrete sweat, dark cells that help store substances for bioactivity, and myoepithelial cells that are involved in structural support and fluid balance [9]. The duct cells reabsorb sodium and chloride ions in the proximal region to prevent the loss of these ions resulting in the secretion of more hypotonic fluid to maintain equilibrium within the circulatory system [9].

Upon stimulation by a variety of factors including elevated body core or skin temperature or some psychogenic stimuli [9], the neurotransmitter acetylcholine is released from sympathetic cholinergic nerve fibers and binds to muscarinic receptors on clear cells thereby triggering calcium release from their intracellular stores [9]. The resultant increase in the clear cell’s cytoplasmic calcium promotes an efflux of potassium chloride causing the clear cells to shrink [9]. Associated with this shrinkage, chloride, potassium, and sodium enter the cell thereby creating a favorable gradient for the subsequent efflux of chloride and potassium into the lumen [9]. The secretion process culminates with osmotically driven water entering the sweat gland lumen with the chloride-rich fluid volume constituting the initial phase of sweat or primary sweat [9]. In its journey from the secretion from clear cells to its ultimate excretion on the skin surface, chloride and sodium primary sweat experiences changes in its chloride and sodium concentration via luminal cell reabsorption of much of the originally secreted ions [9]. This occurs due to the reabsorption actions of cystic fibrosis transmembrane conductance regulator (CFTR) channels and epidermal sodium channels (ENaC) [9]. The hormone aldosterone largely influences the sodium reabsorption process [30].

Eccrine sweat gland functions

Eccrine glands produce sweat in response to hot environments or exercise and can also produce sweat in response to emotional stimuli, such as fear, anxiety, and pain [26]. Although eccrine gland activity is directly controlled by the central nervous system, which regulates sweat secretion by detecting changes in body temperature, they can also be influenced by local cutaneous thermal conditions [27]. It is well documented that eccrine sweat glands are stimulated by cholinergic and adrenergic agonists, however, it has also been shown that a number of different neurotransmitters and humoral agents, such as aldosterone, vasoactive intestinal peptide (VIP), nucleic acids, and estrogen also influence sweat gland function [26]. Similar to many biological mechanisms, neurotransmitters and humoral agents stimulate intracellular signaling pathways which lead to sweat gland activation followed by its production and secretion of sweat [9].

As sweat is produced, e.g., during mild physical exercise, gross activation of eccrine gland activity leads to sweat secretion initially in the form of water vapor and eventually visible liquid perspiration [9,28]. The event is associated with an initial drop in skin temperature and activation of skin surface water loss and stratum corneum moisturization [28]. At first, a variable amount of sweat glands release sweat in vapor form, without the materialization of liquid sweat [28]. Thereafter, liquid sweat is produced by a progressive expansion of active sweat pores [28]. The coalescence of sweat vapor production, liquid sweat production, and stratum corneum moisturization is evident throughout the course of sweat gland activation and is variable among anatomical locations and between individuals [28].

Eccrine sweat gland activation and deactivation

Sweat glands play a role in thermoregulation by helping to maintain proper internal temperature and help defend against hyperthermia and heat exhaustion [31]. It is not fully understood whether sweat glands are constitutively active or if they are activated only at a certain temperature threshold [1,10,31]. An environmental temperature of 30°C has been reported as a threshold for sweat gland activation [32,33]. It has been reported that if the room temperature is less than 20°C and skin temperature is below 30°C, then thermal sweat gland activity is unlikely [34]. However, other reports posit that sweat glands are constitutionally active and secreting continuously [9]. A third report suggests that eccrine glands secrete sweat intermittently with periodic discharges and pauses ranging from about 0.3 to 12 discharges/minute that depend on individuals, circumstances, and body areas [28]. Since eccrine gland activity is increased in a variety of conditions, measurements made during a period of their activation will be affected by an unknown amount [1]. Hence, studies that do not restrict such activation tend to observe greater TEWL values [1,10,17,18].

Attempts to deactivate eccrine glands

Attempts to deactivate eccrine glands can be accomplished by using anticholinergic agents, such as scopolamine [35,36]. Scopolamine decreases the influence of sweat on TEWL values due to its antagonistic effect on muscarinic receptors [35,36]. Muscarinic receptors are G-coupled protein receptors predominantly involved in parasympathetic activity but having sympathetic activity on sweat glands [37]. Normally, muscarinic receptors react to acetylcholine during sympathetic activation and cause sweat secretion. Scopolamine inhibits the receptor from secreting sweat regardless of the presence of acetylcholine [36,37]. While attempting to measure insensible water loss, scopolamine is a generally accepted way to exclude the sweat contribution while still assessing skin barrier function. In the case of exercise, the magnitude of the effect scopolamine exhibits is well illustrated by a study of volar forearm TEWL values in 42 subjects [17]. This study inactivated forearm sweat glands with topically applied scopolamine prior to a three-minute cycling exercise protocol and compared TEWL values between scopolamine treated and untreated forearms [17]. Prior to exercise, untreated and treated TEWL values were similar, being (mean ± standard deviation) respectively 6.50 ± 2.15 g/m^2^/h vs. 6.25 ± 1.03 g/m^2^/h. After exercise, the untreated forearm TEWL value rose to 48.29 ± 18.67 g/m^2^/h whereas the treated forearm TEWL value was essentially unchanged at 6.56 ± 2.15 g/m^2^/h [17].

Another anticholinergic agent, oxybutynin, has been used to try to inactivate sweat glands in a randomized, double-blind, placebo-controlled, cross-over study [38]. In this study, eight healthy individuals with an average age of 35.1 years old (22-50 years) completed a series of exercises with increasing intensity after pre-treatment of either oxybutynin or placebo tablets [38]. Baseline pre-exercise TEWL values for placebo and oxybutynin treatment on the forearm were similar (8.6 ± 3.9 g/m^2^/h vs. 9.4 ± 8.0 g/m^2^/h) as were values on hand palm (45.8 ± 12.1 g/m^2^/h vs. 46.8 ± 14.2 g/m^2^/h) [38]. After maximum intensity exercise for six minutes, post-exercise TEWL values for the forearm rose to 59.4 ± 8.3 g/m^2^/h vs. 61.0 ± 12.9 g/m^2^/h and to 63.1 ± 11.8 g/m^2^/h vs. 64.9 ± 13.3 g/m^2^/h with no significant difference between placebo and oxybutynin treatment outcomes [38]. Thus, the effectiveness of oxybutynin as a useful sweat gland blocker was not supported by these results [38]. Although oxybutynin did not impact TEWL in these healthy persons, when it was taken by 14 patients with hyperhidrosis, 11 of them reported improved quality of life [38,39].

TEWL measurement guidelines

Earlier guidelines for measuring TEWL have been updated by the European Group for Efficacy Measurements on Cosmetics and Other Topical Products (EEMCO) [8,34,40]. Procedural recommendations include an acclimatization period of at least 15-30 min at an ambient temperature (20-22°C) and relative humidity (40-60%) prior to measuring TEWL and at least 20 minutes for skin hydration to eliminate sweating [40]. Various anatomical locations where measurements are effectively taken include the forearm, upper arm, thigh, chest, abdomen, and upper back [40]. In addition, there are suggestions for subjects to avoid using topical lotions or oils on skin parts that are going to be measured and for subjects to avoid exercise and caffeine consumption at least three hours prior to being measured [40]. Although these, and other precautions, are important actions to take prior to recording TEWL values, guidelines do not consider or recommend inactivating sweat glands prior to measurements [41]. This raises issues about the potential confounding effect of unknown levels of sweat gland activation in previous TEWL measurements [2].

Eccrine gland activating factors potentially impacting TEWL measurements

There is a myriad of elements that have been shown to impact TEWL measurements. Endogenous factors are components that alter eccrine gland activity within the body, such as the sympathetic nervous system, emotion, and physical activity [29]. Exogenous factors are aspects that influence the external environment, such as ambient temperature and humidity, in which measurements are taken that impact eccrine gland activity levels [29]. Although it may be theoretically possible to control all these factors, the difficulty in doing so results in only a few being controlled during most TEWL measurements [1]. These factors are discussed individually in the following paragraphs.

Sympathetic nervous system and emotional stress as factors

Sweat production is mainly controlled by the sympathetic nervous system in which postganglionic sudomotor fibers release acetylcholine as their main neurotransmitter that acts on eccrine gland muscarinic receptors [42]. Eccrine glands also have alpha and beta-adrenergic receptors, but adrenergic receptor-induced sweat is only about 10% of that caused by cholinergic stimulation [18]. It is likely that having one’s TEWL measured can itself induce an anxiety-like emotional response in some people and that such emotional sweat impacts TEWL values [17,19]. In one study, forearm TEWL values were obtained in 44 subjects prior to an exercise protocol, and it was found that six subjects (13.6%) demonstrated emotional sweating on the arm if not pre-treated with scopolamine [17]. Psychological sweating in response to emotive stimuli like stress, anxiety, fear, and pain can occur at almost any anatomical site but is most evident on the palms, soles, face, and axilla [19]. The eccrine gland activation and resulting sweating is a result of stimulation of the cholinergic and sympathetic nervous systems triggered by higher brain centers [37].

Physical activity and exercise as factors

Sweating response during exercise not only involves changes in thermal factors like internal and external body temperatures, but also non-thermal factors such as the central nervous system, sympathetic nervous system, mechanoreceptors, metaboreceptors, baroreflex, and more [14]. Furthermore, sweat gland activation in response to passive thermal heating is predominantly impacted by thermal factors and it has been reported that exercise results in a greater overall increase in the number of activated sweat glands, sweat gland output, and sweat gland secretion rates [14]. Pre- and post-exercise measurements have been used to evaluate the effects of eccrine gland sweat production on TEWL [17,29,38]. In a previously mentioned study, the exercise induced increase in forearm TEWL was nearly seven-fold, mainly attributable to the eccrine activation process [17]. Similar results were reported in a group of eight patients with a median age of about 35 years [38]. In that study, pre-exercise TEWL values on the central part of the forearm were 8.6 g/m^2^/h that increased to 59.4 g/m^2^/h after six minutes of cycling [38]. Another study measured TEWL at 14 different anatomical sites pre- and post-exercise to obtain an overall estimate of the average TEWL increase attributable to sweat gland activation [29]. Their results were similar to the previous study findings where there was a significant increase in TEWL, with fluctuation between anatomic sites [29].

Relationship between TEWL and sweat gland density and activation

Eccrine gland density varies considerably among different skin regions and between individuals [29]. Beyond 2-3 years old, the total number of eccrine glands remains the same. Moreover, when humans grow in length, width, and circumference, the eccrine gland density will diverge in certain anatomical locations and accumulate in others [32]. The volar foot surface has the greatest density, followed by the forehead, palms, and lastly the posterior thigh with the lowest density [29]. Since eccrine gland density varies across body regions, it is likely that interregional variations in sweat gland secretion rates are the result of these differences [19,29]. During passive thermal heating and exercise stimulations, eccrine glands from different regions eject sweat at vastly different rates, both across and within individuals [29]. For example, inter-individual, maximal glandular sweat flows ranging from 4 to 34 ug/gland/minute and regional flows of 15.8 ug/gland/minute (forehead), 11.5 ug/gland/minute (forearm), and 17.9 ug/gland/minute (back) [29]. The variations in sweat gland secretion rates at different anatomical regions may relate to changes in TEWL values at these regions. One study suggests that differences in sweat gland function between individuals also contribute to interregional variations in sweat gland secretion, in addition to variations in densities across anatomic locations [43]. From this evidence, it is posited that regional variations in secretion can be attributed to both anatomical and physiological variations within and between individuals [29,43]. Moreover, this study found that some glands do not remain constantly active within a region throughout the sweat gland activation cycle [43]. While the number of active glands may be increasing with thermal loading, these are not always the same glands, with some even decreasing their activity over time. This concept is observed in the table below [29]. An extensive compilation of literature data detailing TEWL values and sweat gland density in different body segments is attributable to the comprehensive work of Taylor NA and Machado-Moreira CA [29] and Pinnagoda J, Tupker RA, Agner T, Serup J [34]. Selected data derived from their work is summarized in Table 1.

**Table 1:** Basal and activated* TEWL values, sweat gland densities, and sweat gland rate of secretion at some often-measured skin sites Basal and activated* TEWL values (g/m^2^/h), sweat gland densities (glands/cm^2^), and sweat gland rate of secretion (g/m^2^/h) at some often-measured skin sites. Values are rounded to the upper whole number. *These values were approximated due to the interpretation of data in graph form. *Mechanism of activation for activated values of TEWL and sweat gland density accomplished with light-moderate intensity exercise (cycling). TEWL = transepidermal water loss References: Basal TEWL values retrieved from Pinnagoda [34], activated TEWL values, basal and activated sweat gland density values, and basal and activated sweat gland rate of secretion retrieved from Taylor [29]. Author's own creation.

Anatomical Site	TEWL		Sweat Gland Density		Sweat Gland Secretion Rate	
	Basal	Activated*	Basal	Activated*	Basal	Activated*
Forehead	17	55	155	220	600	2,040
Forearm - Anterior	6	30	108	120	180	540
Hand - Palm	48	90	241	520	180	900
Hand - Dorsum	7	58	176	170	300	1,200
Leg - Medial	6	30	114	60	120	480
Foot - Sole	27	60	294	500	150	300
Abdomen	6	30	141	100	210	600
Upper Back	6	30	106	100	360	1,020

Age as a factor

The determination and interpretation of the impact of aging on TEWL are complicated by concomitant skin changes sometimes associated with chronological and photoaging skin [44]. If changes in both stratum corneum and TEWL occur with aging, interpreting the role of TEWL changes in barrier function is unclear [44]. There have been few studies that have directly compared younger and older age groups [44,45]. One evaluated forearm TEWL in 26 younger (19-42 years) vs. 18 older (69-95 years) and reported no significant difference in baseline TEWL values [44]. After these subjects were exposed to 24 hours of local forearm occlusion with polypropylene chambers, TEWL increased in both groups but the recovery toward baseline TEWL values was significantly delayed in the older group [44]. Another small study consisted of seven young adults and eight older persons with mean ages of 25.9 and 74.6 years respectively in whom TEWL was measured at multiple sites [45]. They reported that although mean values of TEWL tended to be somewhat less in the older group, most were not statistically significant. For example, they reported mean volar forearm values of 5.0 vs. 2.3 for young vs. older groups as being nonsignificant whereas upper arm values of 3.8 vs. 1.8 were significant (p<0.05). At the other end of the age spectrum, TEWL values measured on the forearms of over 1000 newborns within 96 hours of birth yielded an overall mean and SD of 7.06 ± 3.41 g/m^2^/h when measured with an open system [46]. Measurements on 104 neonates within 72 hours of birth using a closed TEWL system yielded a higher value of 13.4 ± 5.7 g/m^2^/h [47]. Measurements of forearm TEWL in 150 women ranging in age from 18 to 80 years found no correlation between TEWL and age with values ranging from 8.81 to 9.55 g/m^2^/h [48]. Similar measurements in 150 men ranging in age from 20 to 70 years revealed mean values ranging from 5.43 to 5.03 from the youngest to the oldest group of 30 men [49]. Contrastingly, a small study that compared TEWL values in 14 young (mean age 26.7 years) vs. 15 older (mean age 70.5 years) subjects reported lower values in the older group [50]. However, another small study of 10 young (24-34 years) vs. 10 older (66-83 years) reported no age-related difference in forearm TEWL values or at any other measured site [51]. Thus, the preponderance of the data suggests that only minor changes in TEWL related to age per se occur at most measured sites, but the matter is not fully settled [44-50].

Ambient conditions as factors

Eccrine glands coordinate with higher brain centers to help maintain temperature homeostasis [52]. While eccrine gland activity increases with increasing temperature, the threshold at which eccrine glands are activated is unclear and some studies suggest they are continuously active with increased activation when temperature increases [1,10,31,53]. A small study examining forearm TEWL values in six healthy women (26-35 years old) at room temperatures of 20°C, 25°C, and 30°C reported a significant positive correlation between TEWL and temperature [11]. TEWL average values increased from about 5 g/m^2^/h to 10 g/m^2^/h to 15 g/m^2^/h respectively [11]. Another study of 16 young adults (18-22 years) in whom TEWL was measured on the dorsal aspect of the forearm reported that TEWL varied directly with three descending ambient temperature intervals between 32-24°C, 28-24°C, and 20-24°C, with the most pronounced change between 32-24°C [54]. Over this range, TEWL decreased significantly from approximately 33 g/m^2^/h to 11 g/m^2^/h after 20 minutes. A room temperature increase from 22°C to 30°C is reported to nearly double the TEWL as measured on the forearm [34]. It appears that ambient air temperature and TEWL have a nearly linear relationship but the relationship between skin surface temperature and TEWL may be logarithmic. In addition to the effects of temperature, humidity has an indirect relationship with TEWL, where increased relative humidity correlates with decreasing TEWL [1,11]. Effects of humidity on TEWL are of concern since it has been reported that even in low humidity conditions sweating can persist although the skin surface appears totally dry [29]. Therefore, ambient temperature and humidity in the laboratory or clinical environment in which TEWL measurements are being made are a minimum needing adequate control and reporting [29]. In addition to environmental and ambient temperature effects on TEWL, it has been shown that local skin heating can have a dramatic effect on TEWL [55]. In this study, volar forearm skin was locally heated to 40-42°C for 12 minutes and changes in TEWL were measured in 30 healthy adults [55]. Mean and SD pre-heat TEWL values of 8.8 ± 1.8 g/m^2^/h rose to 39.6 ± 23.4 g/m^2^/h and remained elevated for 18 minutes post-heating. This TEWL increase is accompanied by an increase in stratum corneum hydration in a manner similar to that reported for whole-body heating [56].

Environment and habitat as factors

Permanent residents of tropical climates are able to tolerate heat better than those from more temperate climates [20]. Several factors contribute to this including sweat gland density, sweat output per gland, and responsiveness to sweat-inducing stimuli [20,32]. A comparison of forearm sweat gland responses to acetylcholine between men from a temperate climate (20 healthy Japanese males) and those from a tropical climate (10 healthy African males) has shown differences [32]. Using iontophoresis, sweat produced by acetylcholine was measured while subjects were seated in a climate-controlled room at 24°C [32]. Results indicated that sweat gland sudomotor responses to acetylcholine stimulation and the acetylcholine-induced TEWL values were greater in Japanese men from temperate environments than in men from tropical climates of Africa [32]. Sweat gland density, measured by an iodine-impregnated paper method, was 50.6% higher in Japanese men vs. African men (p<0.001) and sweat gland output was 20.4% higher (p<0.001) in the Japanese men as assessed via TEWL values [32]. The authors speculated that this difference was possibly due to those living in temperate climates being less adapted to hotter climates [32]. These results point to the potential importance of the origin of the subject, their ancestry, and their predominant early life environment as factors to be considered for TEWL assessments and comparisons [32]. In contrast to researching the subject’s intrinsic cool-down ability, another study induced heat acclimatization over a 10-day period by having subjects submerge their legs in a warm tub of water for 2 hours at a time [20]. This study introduced an extrinsic means of TEWL being altered [20]. The results at the end of the study showed that these heat-acclimated subjects had increased TEWL values from a short-term standpoint [20].

Eccrine gland contributions to TEWL values in hyperhidrosis

Hyperhidrosis is defined as excessive sweating [21] and is present in various conditions including primary palmoplantar hyperhidrosis [23,24], overall body hyperhidrosis [21], and cystic fibrosis (CF) [22]. Comparisons of TEWL values between such patients and non-affected persons can shed light on the impact of eccrine gland contribution to TEWL values. One such study evaluated and compared TEWL values (g/m^2^/h) on hand palmar and foot plantar skin in 50 patients with hyperhidrosis and 25 control subjects [23]. TEWL values were greater on patient hands (133.6 ± 51.0 vs. 37.9 ± 18.4, p<0.001) and feet (71.8.6 ± 40.3 vs. 27.6 ± 14.3, p<0.001). TEWL measurements were also done in a group of 35 control subjects and 45 patients with hyperhidrosis before and after the patients had a bilateral thoracoscopic sympathectomy (BTS) [24]. Before BTS, patient hand TEWL (g/m^2^/h) was greater than the control’s (142.7 ± 43.6 vs. 115.8 ± 48.7, p<0.0001), and greater on feet (87.5 ± 28.8 vs. 57.7 ± 24.7, p<0.0001). After BTS, palmar TEWL decreased to 49.1 ± 29.8, p<0.0001, whereas plantar TEWL did not significantly change [24]. In contrast to the greater TEWL values at palmer and plantar sites of persons with hyperhidrosis vs. controls, there was no TEWL difference when measured at the lumbar region that has few eccrine glands [24]. TEWL values for 20 persons with and without hyperhidrosis averaged between 10-12 g/m^2^/h [21]. However, palmar hand values were significantly higher in the hyperhidrosis patients (123.5 g/m^2^/h vs. 46.4 g/m^2^/h, p<0.001). Similar findings as this have been reported [57].

Eccrine gland contributions to TEWL values in CF

Additional insight into the role of eccrine glands in measured TEWL values comes from TEWL value comparisons between 51 children with CF and 25 children free of this condition [22]. CF is a genetic disorder with dysregulation of chloride channels, leading to hyperhidrosis [22]. The CF patients vs. the control group had a significantly greater median TEWL (282.4 g/m^2^/h vs. 173.1 g/m^2^/h, p<0.001). These findings suggest that increased eccrine activity likely contributes to the increased TEWL in these patients [22].

## Conclusions

The present findings indicate a multiplicity of biological and environmental variables impacting eccrine gland activity and thereby potentially affecting measured TEWL values. Factors include those related to sympathetic system activation, emotion-provoking stimuli, physical activity, eccrine gland density and sensitivity, age, sex, and environmentally related factors including temperature, humidity, and even a person’s habitat of living. The exact contribution of eccrine sweat gland activity to measured TEWL values is currently unknown. This is partly because the contribution varies greatly with the aforementioned factors. Even if laboratory conditions adhere to EEMCO guidelines and other recommendations, it is not yet possible to separate the eccrine activation component from the parameter of true interest in the assessment of the skin’s physiological barrier function except for full gland deactivation.

The amount that such eccrine gland activation impacts the measured value of TEWL is generally not determined using currently available methods and the only sure way to eliminate a confounding effect is to inactivate the glands during such TEWL measurements. Because such eccrine gland deactivating approach is not usually desirable or even possible, other approaches would be recommended. One would be the development of a measuring device that could distinguish between the component of TEWL that is associated with the skin barrier function and the other that is attributable to sweat gland activation. Further research and development along these lines appear warranted.
